# Anatomical Study of SCIAP (Superficial Circumflex Iliac Artery Perforator) and SIEA (Superificial Inferior Epigastric Artery) Flaps for Flap Harvest Training in the Swine Model

**DOI:** 10.1002/micr.70056

**Published:** 2025-03-25

**Authors:** M. d'Alessio, S. Avvedimento, S. Castaldo, V. Manfellotto, D. Ciclamini, E. Grella, G. F. Nicoletti, P. Tos, S. D'Arpa

**Affiliations:** ^1^ Plastic and Reconstructive Surgery Unit, Multidisciplinary Department of Medical‐Surgical and Dental Specialties University of Campania Luigi Vanvitelli Naples Italy; ^2^ Plastic and Reconstructive Surgery Department Cardarelli Hospital Naples Italy; ^3^ U.O.C. Ricerca Formazione and Cooperazione Internazionale A.O.R.N. “Antonio Cardarelli” Naples Italy; ^4^ Department of Orthopaedics and Traumatology, Hand and Reconstructive Microsurgery Unit Orthopaedic Trauma Center CTO “A.O.U. Città Della Salute e Della Scienza” Turin Italy; ^5^ Hand Surgery and Reconstructive Microsurgery Unit Orthopedic Institute G. Pini‐CTO Milan Italy; ^6^ Plastic and Reconstructive Surgery La Maddalena Cancer Center Palermo Italy

## Abstract

**Introduction:**

Microsurgery training necessitates a progression from basic to advanced techniques, utilizing artificial models, cadaver labs, and live animals. Living animals are of paramount importance to simulate flap harvest and have a real‐life experience with immediate feedback on the quality of dissection. Pigs are effective models for flap harvest training since the anatomy is comparable to that of humans, and so are many flap models. This study introduces the superficial circumflex iliac artery perforator (SCIAP) and superficial inferior epigastric artery (SIEA) flaps in pigs.

**Materials and Methods:**

Dissections were performed on 10 female swine ( *Sus scrofa domesticus* , ssp. Large white; 35–40 kg) during a perforator flaps dissection course organized by the Italian Society for Microsurgery (SIM: Società Italiana di Microchirurgia). Adhering to ethical guidelines and the 3R principles, animals were anesthetized and euthanized humanely post‐procedure. Twenty SCIAP and 20 SIEA flaps were harvested: vessel anatomy and presence, anatomical landmarks, pedicle caliber and length, flap viability, and design were evaluated.

**Results:**

The SCIAP and SIEA were present on both sides in all animals (100%). The SCIAP was constantly found between 4 and 6 cm medially to the ASIS. After giving off the SCIAP, the SIEA always arose from the SCIAP continuing medially toward the abdomen, giving off small branches (4–8) to the skin. Mean pedicle length (distance between origin from the femoral vessel end entry into the flap) was 8.035 cm for the SCIAP artery (SD ± 0.09), 8.04 cm for the SCIAP vein (SD ± 0.11), 14.98 cm for the SIEA artery (SD ± 0.10), and 14.98 cm for the SIEA vein (SD ± 0.24). Mean arterial caliber was 2.201 mm for the SCIAP (SD ± 0.24) and 1.89 mm for the SIEA (SD ± 0.217). Mean vein caliber was 2.23 mm for the SCIAP (SD ± 0.18) and 2.14 mm for the SIEA (SD ± 0.162). In total, 20 SCIAP and 20 SIEA flaps were harvested. Two of them (one SCIAP and one SIEA) showed signs of hypoperfusion with a 95% viability rate. The SCIAP flap was located 4–6 cm medial to the ASIS, while the SIEA was found along a line connecting the ASIS and the midpoint of a line connecting the second and third nipples.

**Conclusion:**

The SCIAP and SIEA flaps in pigs offer a valuable addition to microsurgical training, replicating important human flaps. Their consistent anatomy and the ability to harvest them in different positions enhance their utility in training programs. These findings support the integration of these flaps into pig model courses, optimizing animal use and training efficacy in line with the 3R principles.

## 1 Introduction

Microsurgery training needs practice from basic techniques to advanced anastomosis, from flap anatomy to proficient flap harvest. Hands‐on training in animals and cadavers allows to practice and sharpen the essential skills needed outside the clinical setting before applying techniques on patients (Loh et al. 2018). Microsurgery schools nowadays include artificial models, cadaver labs, and live animals in their training programs. The Italian Society for Microsurgery (SIM: Società Italiana di Microchirurgia) has a five‐step training program that begins with basic microsurgery courses on artificial models, held in different centers all over Italy. The second step is the advanced microsurgery course, which allows the development of advanced microsurgical skills in rat models to achieve consistent proficiency in vessels and nerve anastomosis. The third step is the cadaver lab, to learn human flap anatomy. The fourth step is the perforator flap harvest course on live pigs to learn efficient flap harvest in a living model. The last step is the clinical fellowship: through rotations, students attend five different departments to learn the clinical application of the skills they have acquired over 2 years (Pignatti et al. 2020).

Pigs are an excellent flap harvest training model (Haughey and Panje 1989): they offer several perforator flap models, have an anatomy that is comparable to humans, require a demanding dissection, more difficult than in humans—which is optimal for skills development—and, most importantly, are alive (Stefanidis et al. 2013; Daniel and Kerrigan 1982). Compared to cadavers, a living model provides immediate feedback on the quality of dissection: flap vascularization and survival can be immediately verified. Consequently, technique must be fine, tissue handling must be delicate and precise; otherwise, the flap will not be vascularized if the vessels are damaged.

Several flap models exist. The SIM perforator flap course program includes the harvest of the deep superior epigastric artery perforator (DSEAP) flap (Pignatti et al. 2020) (model for DIEAP flap harvest), internal mammary artery perforator (IMAP), internal mammary vessels (IMV) preparation (Nistor et al. 2013a; Cajozzo et al. 2018), lateral intercostal artery perforator (LICAP) flap (Nistor et al. 2022), superior gluteal artery perforator (SGAP) flap (Favuzza et al. 2018), thoracodorsal artery perforator (TDAP) flap, and dorsocervicalis flap (Nistor et al. 2013b; Murphy and Sonntag 1998). Students work simultaneously in teams of two, each team using one side of the animal. In 2 days, they manage to perform all the above‐mentioned flaps, maximizing the use of animals following the reduction principle of the 3R (Replacement, Reduction, Refinement) concept (MacArthur Clark 2018). The model has been published before (Pignatti et al. 2020).

There is a need for constant improvement of animal training by finding all flaps that reproduce the flaps found in humans to optimize resources and reduce the number of animals needed (3R concept) (MacArthur Clark 2018).

As is often the case with new findings, recognizing a superficial circumflex iliac artery perforator (SCIAP) was incidental during SGAP flap dissection (Bodin et al. 2015). The skin island of the SGAP is described transversely to a line connecting the greater trochanter (GT) and the anterior superior iliac spine (ASIS) (Favuzza et al. 2018), with this line crossing the flap. During dissections performed this way, a septal perforator was constantly found in the anterior half of the flap (anterior to the above‐mentioned line). This perforator was recognized as potentially being correspondent to the SCIAP flap pedicle, and its anatomy was investigated.

In this article, the anatomy and the harvesting technique of the SCIAP and SEAP flaps in pigs are described, filling the gap due to the lack of these important flaps.

## 2 Materials and Methods

Dissections were performed during the perforator flaps dissection course on pigs at UOC Formazione, Ricerca e Cooperazione internazionale, Pad. X, AORN A. Cardarelli, Napoli, organized by the Italian Society for Microsurgery.

Ten female swine ( *Sus scrofa domesticus* ssp., large white; 35–40 kg) were used for the dissections. The animals were included in an experimental protocol (protocol no. 38.2010.01.001), which received approval from the local Ethical Committee on Animal Experimentation.

Following the Italian law (D.lgs 26/2014) (Gazzetta Ufficiale della Repubblica Italiana 2014), the “3R rules” were respected: (a) replacement: nonanimal alternatives cannot be used in this type of experiment since bleeding is needed to recognize an adequately performed surgery and tissue manipulation (Gazzetta Ufficiale della Repubblica Italiana 2014); (b) reduction: the lowest number of subjects to ensure adequate power for our study was used; (c) refinement: all procedures were performed under laboratory animal science experts' supervision to avoid, minimize, and alleviate animal distress.

### 2.1 Surgical Procedures

General anesthesia was administered as follows (following the Italian law: D.lgs 26/2014): premedication with an intramuscular injection of zolazepam–tiletamine (0.5 mL/kg), induction with propofol EV (6 mg/kg) and ketamine (10 mg/kg) (IV) intravenous, maintenance with inhaled sevoflurane 2%, rocuronium (0.2 mg/kg), and propofol infusion if needed. Analgesia was achieved with butorphanol (0.1–0.4 mg/kg) IV. Orotracheal intubation, ventilation with an anesthetic machine (MINDRAY Wato Ex‐35vet), and multiparametric monitoring (MINDRAY ePM 12 M Vet) were utilized. At the end of the procedure, all animals were euthanized with an intravenous injection of a lethal dose of embutramide–mebezonium iodide–tetracaine (1 mL/3 kg).

Pigs were placed in the lateral decubitus position during flap dissection. All the procedures were performed using 2.5× and 4.5× loupe magnification. Differences between pigs and humans were evidenced and reported for each flap.

#### 2.1.1 Superficial Circumflex Iliac Artery Perforator (SCIAP) Flap

**FIGURE 1:**
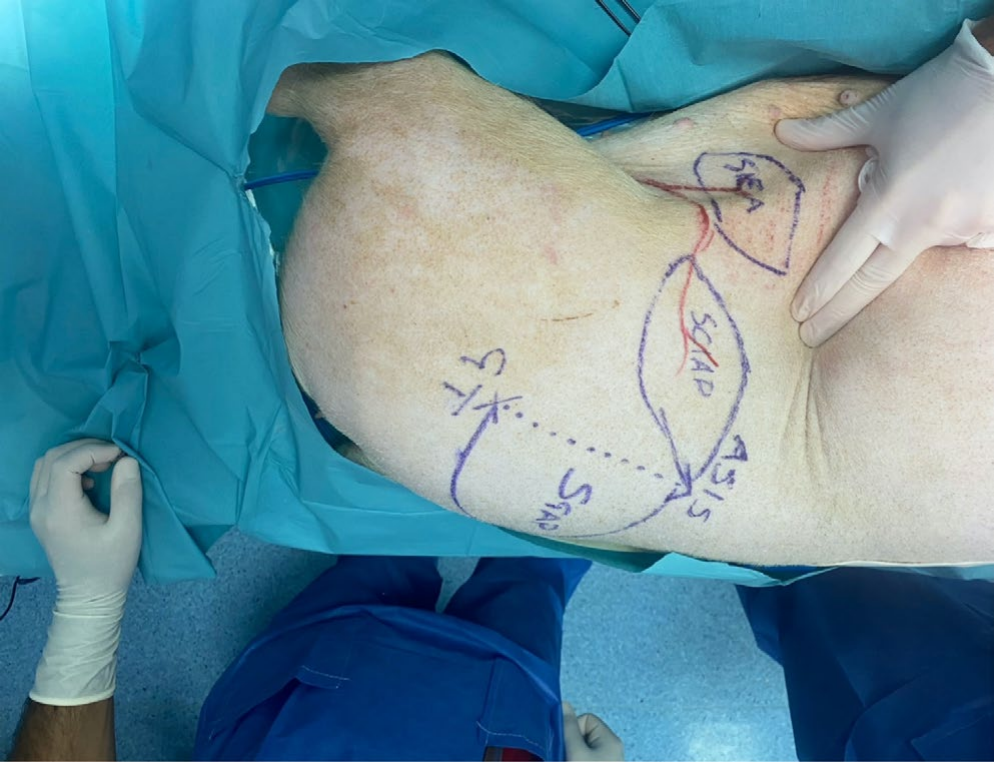
Bird's eye view of the pig in the right lateral decubitus position. The head is on the right‐hand side; the posterior is at the bottom of the picture. The SCIAP and SIEA flaps are marked as described in the text. The SGAP flap is also shown, drawn and harvested only posteriorly to the line connecting the anterior superior iliac spine (ASIS) and the greater trochanter (GT). Likewise in humans, the SCIAP points in the direction of the ASIS. The hand pressing the skin shows the skin fold formed by the panniculus carnosus (see the text for details) between the index finger and the thumb.

Landmarks for the SCIAP flap were the ASIS and the groin crease (Figure 1).

The flap was drawn as an ellipse, placed cranially and parallel to the groin crease, with one end at the ASIS. It differed from humans since placing the island beyond the ASIS in pigs would overlap with the territory of the SGAP flap, which had to be preserved for dissection (Nistor et al. 2022).

The caudal edge of the flap was incised first. After the panniculus carnosus was divided, the equivalent of the human quadriceps femoris was visualized, and dissection continued on top of its fascia towards the inguinal ligament until the SCIAP was visualized as it entered the panniculus carnosus to eventually reach the skin. At this point, perforator dissection continued to the femoral vessels' origin. Before the femoral vessels were reached, the SIEA emergency was met. Several sensory nerves were crossed during dissection and needed to be isolated and preserved (resulting in good exercise for training surgeons).

#### 2.1.2 Superficial Inferior Epigastric Artery (SIEA) Flap

**FIGURE 2:**
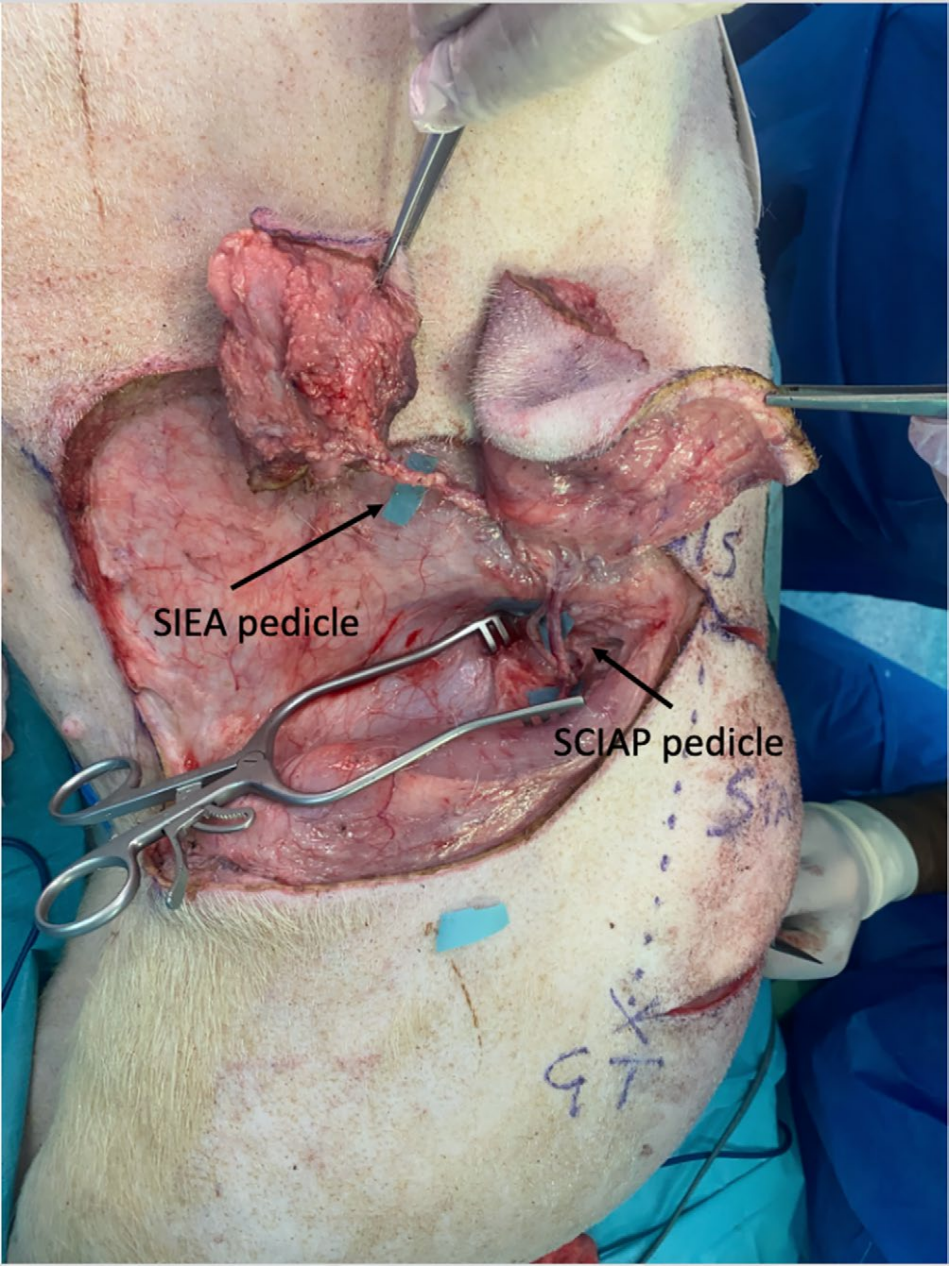
Bird's eye view (posterior on the right‐hand side, cranial top of the picture) after flap harvest has been completed. The SCIAP and SIEA have been harvested as a chimeric flap. The three backgrounds indicate the SIEA pedicle (top left), the SCIAP pedicle (top right) and the Quadriceps femoris equivalent pedicle (bottom right).

**FIGURE 3:**
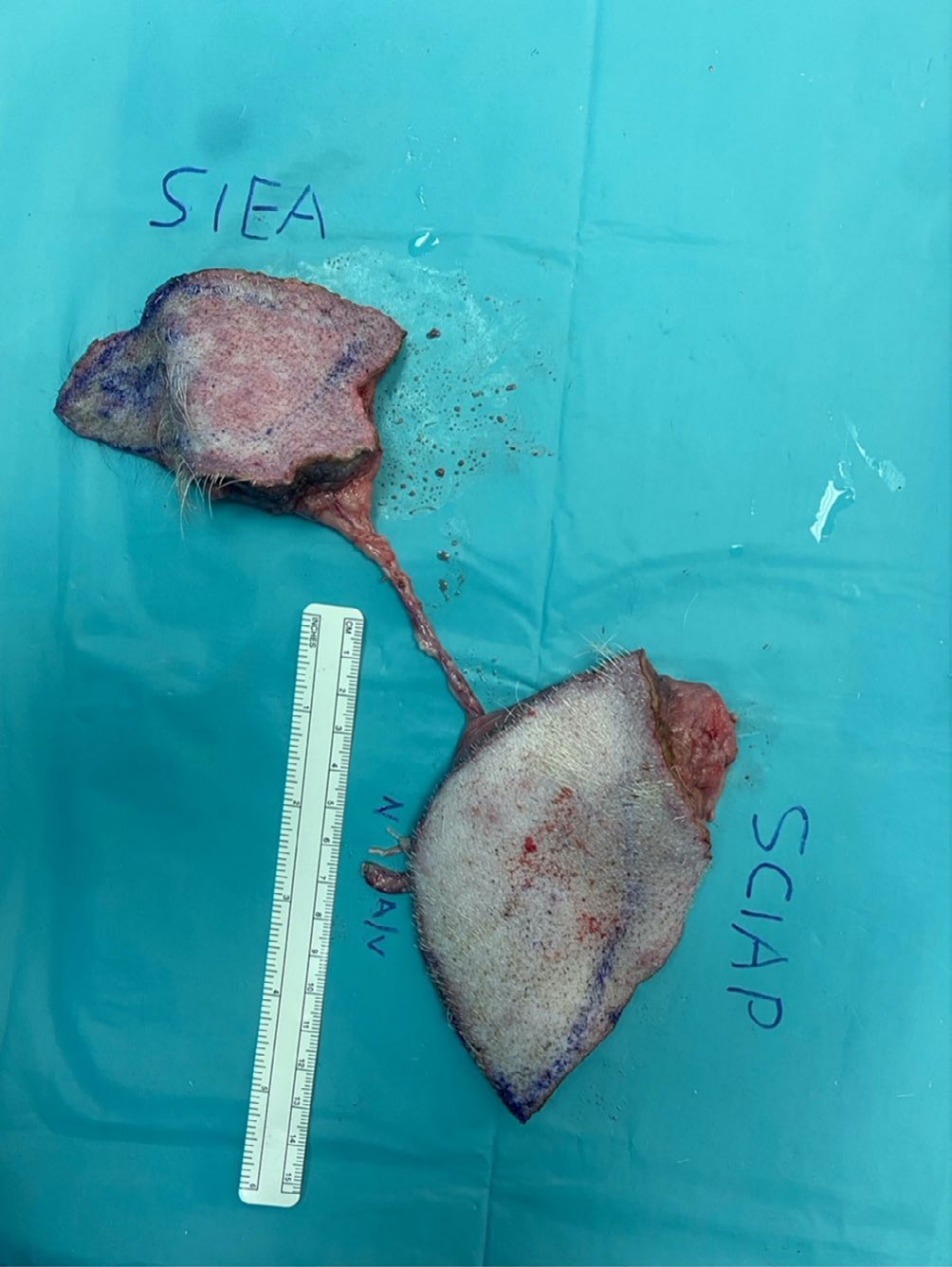
The chimeric flap composed of a SCIAP and a SIEA flaps after pedicle division. The SIEA flap pedicle is constantly longer than the SCIAP flap pedicle (see the text for details).

**FIGURE 4:**
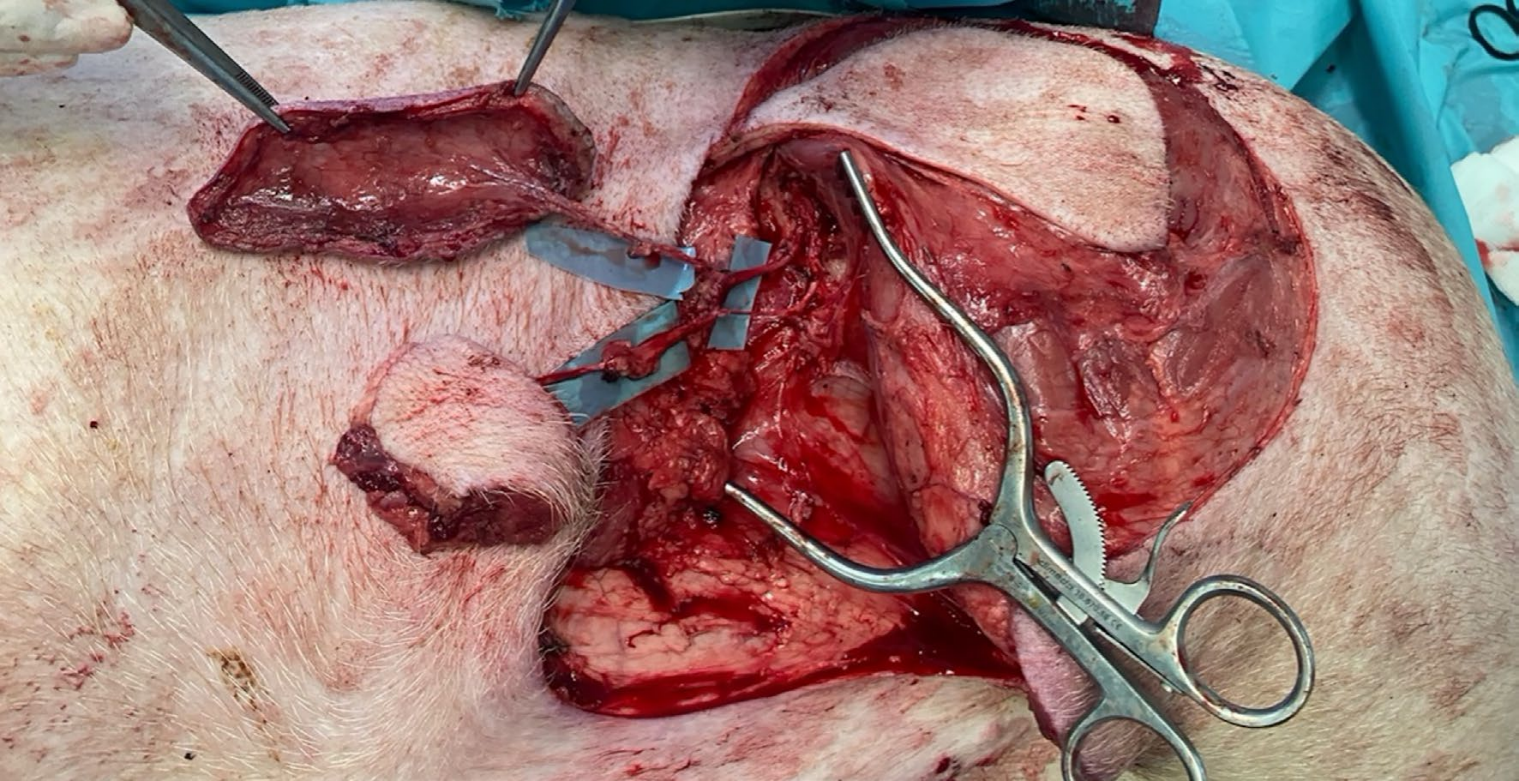
Anterior view (head on the left‐hand side, from the position of the surgeon) that harvests the SCIAP and SIEA flaps at the end of the dissection. While one surgeon harvests the SCIAP and SIEA flaps, the other is able to harvest a SGAP flap successfully.

Landmarks for the SIEA flap were the groin crease and the skin fold that forms at the level of the equivalent of the human linea semilunaris (Figure 1). This fold forms since the panniculus carnosus thins out to almost disappear in the groin and pubic region. This line is the axis of the SIEA, and the flap was drawn as a transverse ellipse. All perforators and branches of the SIEA were found around a line connecting the ASIS and the midpoint between the second and third nipple (starting caudally). The caudal edge was incised first. After the division of the panniculus carnosus, branches of the SIEA were visualized lying on top of the anterior rectus sheath. The vessels were skeletonized and followed until their common origin from the SCIA and then to the femoral vessels, and both flaps can be harvested as a chimeric flap on a single pedicle (Figures 2, 3, 4).

### 2.2 Variables Measured

Feasibility of flap harvest, flap viability, anatomical landmarks, pedicle length and diameter, perforator location, and harvesting technique were assessed.

## 3 Results

Feasibility of flap harvest: The SCIAP and SIEA were present on both sides in all animals (100%). In total, 20 SCIAP and 20 SIEA flaps were harvested. All flaps were dissected and harvested.

Flap viability: 95%. In two different animals, one out of 20 SCIAPs flaps and one out of 20 SIEAs flaps showed skin discoloration due to hypoperfusion. A supposed inadvertent pedicle injury could have been the cause of these complications.

Anatomical landmarks: The SCIAP was constantly found between 4 and 6 cm medially to the ASIS. After giving off the SCIAP, a large branch continued medially toward the abdomen, giving off small branches (4–8) to the skin. This was considered the corresponding vessel of the SIEA. Likewise, in humans, SCIA and SIEA arise from a common branch of the femoral artery that soon divides into two vessels (Aydin and Nasir 2007). In pigs, the common branch was longer than in humans. The SCIAP emerged from the femoral vessels, together with a large branch for the quadriceps femoris muscle. Unlike humans, no superficial veins were found in pigs, and veins exclusively arose from the femoral vein and ran as venae comitantes.

The SCIAP pedicle was accompanied by nerves for its entire length, possibly sensory, since no muscle contraction was observed during manipulation and nerves were not noticed to enter any muscle. Investigation of nerve anatomy was behind the purpose of this study. Mean pedicle length (distance between origin from the femoral vessel end entry into the flap) was 8.03 cm for the SCIAP artery (SD ± 0.09), 8.04 cm for the SCIAP vein (SD ± 0.11), 14.98 cm for the SIEA artery (SD ± 0.10), and 14.98 cm for the SIEA vein (SD ± 0.24).

Mean arterial caliber was 2.20 mm for the SCIAP (SD ± 0.24) and 1.89 mm for the SIEA (SD ± 0.21). Mean vein caliber was 2.23 mm for the SCIAP (SD ± 0.18) and 2.14 mm for the SIEA (SD ± 0.16) (Table 1).

**TABLE 1:** Flap measurements.

Flap	Mean arterial diameter (mm)	Mean arterial length (cm)	Mean venous diameter (mm)	Mean venous length (cm)
SCIAP	2.20 ± 0.24	8.03 ± 0.09	2.20 ± 0.24	2.23 ± 0.18
SIEA	1.89 ± 0.21	14.98 ± 0.10	2.39	2.14 ± 0.15

Perforator location: all perforators to the SCIAP were found along the groin crease, 4–6 cm medial to the ASIS. All perforators and branches of the SIEA were found around a line connecting the ASIS and the midpoint between the second and third nipples (starting caudally).

Harvesting technique: the two flaps were comfortably harvested while other teams performed the SGAP, TDAP, and dorsocervicalis flaps' dissection in the lateral decubitus position (see Section 2 and Table 1).

## 4 Discussion

This study demonstrates that the SCIAP and SIEA flaps can be consistently harvested in pigs. The anatomy is constant, and the flaps can always be harvested. Two viable flaps can be harvested independently or on the same pedicle as a chimeric flap. The anatomy of pig flaps is comparable to human anatomy in the case of a SCIAP flap based on the superficial branch of the SCIA. Unlike humans (Yoshimatsu et al. 2015; Yano et al. 2021), a deep branch of the SCIA, running on top of the sartorius muscle, is not present in pigs. The description of these flaps introduces an additional exercise to be performed in pigs, allowing training of two important flaps in humans.

These two flap models have several advantages. They can be harvested both in the lateral decubitus and supine positions. Therefore, the SCIAP and the SIEA flaps can be harvested simultaneously with the contralateral DSEAP (Roggio et al. 2018), IMAP, LICAP flaps harvesting or the IMV preparation in the supine position or during SGAP, TDAP, or dorsocervicalis flaps harvesting in the lateral decubitus, allowing for the simultaneous work of two or three teams. This means that this exercise can be added to pig courses without adding extra time since it can be harvested concurrently with other flaps. Animal exploitation is maximized during courses, the 3R principle is respected, and precious time is saved. The pedicles are lengthy, up to 15 cm. Students must perform a lengthy and accurate dissection of a long pedicle, taking care not to damage it, let it dry, and be able to check if their dissection results in a viable flap right away. Although this is not a musculocutaneous perforator dissection, it is an excellent exercise.

Nerves cross the pedicle. Dissection of nerves from the pedicle allows for training of fundamental skills in perforator flap surgery, which is essential in perforator flap harvest since nerve sparing is pivotal to function preservation. The two pedicles join together. This allows practicing both the harvest of a chimeric flap made of two separate skin islands and the harvest of a single, large, bipedicle perforator flap.

There are some limitations to this model. The SCIAP and SIEA flaps in pigs present some differences compared to humans. There is no deep branch of the SCIAP in pigs. The perforators are not musculocutaneous, which is a relative disadvantage. Although the intramuscular dissection is not trained with these flaps, they still simulate the real‐life clinical scenario of a direct cutaneous perforator, which could be found in humans. The superficial and deep fat layers cannot be identified, so the harvest of a thin subdermal flap (above superficial fascia) cannot be trained. This is true for the pig model in general, not only for these flaps. Pigs have a panniculus carnosus and a fat layer which differ from the human fat: lobules and layers are not identifiable in the pig model. A thin flap harvest is a very advanced skill which, to date, can only be developed by operating on humans (Hong et al. 2014; Kim et al. 2015; D'Arpa et al. 2019; Fathi et al. 2008). Cadavers might help in visualizing the anatomy of thin flaps. A relative disadvantage of these models is the difference in anatomy compared to humans. The SCIAP in pigs has a more constant anatomy, does not have the two branches (superficial and deep), and does not have a superficial vein. The SIEA is more constant too and has no superficial drainage. This means that the real‐life scenario of an absent pedicle or the intraoperative choice of a deep or superficial pedicle or a deep or superficial venous drainage is not simulated in pigs. However, the course is for skills training and clinical decision‐making, and intraoperative human anatomy must be trained in the OR. As clarified by Franchi et al. (2024). these two branches can be called SCIAP and SIEA in the absence of a SCIAP deep branch in pigs.

Limitations of this study include the fact that this is a descriptive study. No statistics have been performed. Only some descriptive data are reported. We believe this is a relative limitation since statistics are not needed in an anatomical description. Animal model training is of paramount importance to learn perforator flap harvesting techniques since it provides immediate feedback on the quality of dissection with all the challenges of living tissue surgery but without the risks and consequences of failure in real patients. It offers a virtually unlimited training source that might be exploited until proficiency is reached before starting operations in humans. Finding efficient models for perforator flaps is becoming increasingly important, especially after working hours restrictions for residents have been introduced worldwide and surgical exposure is reduced. In this scenario, an alternative training option must be provided to warrant sufficient surgical exposure to young surgeons. The importance of this study is that it describes two new flaps in pigs, providing more training opportunities to the microsurgeon. Especially in times like these, when working hours are being reduced and so is surgical exposure, increasing potential nonhuman living models is essential (Bergmeister et al. 2020; Ahmed et al. 2014).

Several flap training models in pigs have already been standardized and proven effective in training surgical skills. The inclusion of the SIEA and SCIAP in the exercises during flaps on pig model dissection might offer a valid training opportunity for microsurgical trainees. Although they show differences with their human analogs, like most pig flap models, they offer a good opportunity for skills development. The relevance of these findings lies in the description of two new flaps in pigs that reproduce two very useful and versatile flaps in humans and in the description of the harvesting technique of these flaps that can be harvested simultaneously with other flaps in pigs, allowing for a reduction in the number of animals needed to train, according to the 3R concept in animal experimentation.

## 5 Conclusion

The SCIAP and SIEA can be harvested in pigs. Their anatomy resulted in constant and reproducible outcomes. The possibility of their harvest both in the lateral and the supine positions makes them a versatile model to be included in training courses in the pig model, allowing to add two flaps to the courses without necessarily having to prolong the length of the course by training surgeons simultaneously and optimizing resources. They have been implemented in pigs' SIM perforator flap course and added to the already described sequence of flaps.

## Ethics Statement

The animals were included in an experimental protocol (protocol no. 38.2010.01.001), which received approval from the local Ethical Committee on Animal Experimentation.

## Consent

The authors have nothing to report.

## Conflicts of Interest

The authors declare no conflicts of interest.

## Data Availability

Data sharing not applicable to this article as no datasets were generated or analysed during the current study.
